# Implementation and evaluation of a novel community-based urban mobile health clinic in Toronto, Ontario

**DOI:** 10.17269/s41997-024-00962-x

**Published:** 2025-01-13

**Authors:** Meghan O’Neill, Robert J. Redelmeier, Camilla Michalski, Raymond Macaraeg, Maureen Gans, Alice Schoffel, Lori M. Diemert, Luwam Ogbaselassie, Laura C. Rosella, Andrew Boozary

**Affiliations:** 1 Population Health Analytics Lab, Dalla Lana School of Public Health, University of Toronto, Toronto, ON Canada; 2 University Health Network, Toronto, ON Canada; 3 Gattuso Centre for Social Medicine and Population Health, University Health Network, Toronto, ON Canada; 4 Parkdale Queen West Community Health Centre, Toronto, ON Canada; 5 Laboratory Medicine and Pathobiology, Temerty Faculty of Medicine, University of Toronto, Toronto, ON Canada; 6 ICES, Toronto, ON Canada; 7 Institute for Better Health, Trillium Health Partners, Mississauga, ON Canada; 8 Institute for Health Policy Management and Evaluation, University of Toronto, Toronto, ON Canada

**Keywords:** Social determinants of health, Health equity, Mobile health clinic, Mobile van, Community-based, Intervention, Déterminants sociaux de la santé, Équité en santé, Clinique de santé mobile, Unité mobile, Communautaire, Intervention

## Abstract

**Setting:**

Despite Canada’s single-payer health system, marginalized populations often experience poor health outcomes and barriers to healthcare access. In response, mobile health clinics (MHCs) have been deployed in several cities across Canada. MHCs are well established in the United States; however, little is known about their role and impact in a country with universal healthcare. We describe the implementation of an urban MHC and early learnings from a mixed methods process and outcome-oriented evaluation.

**Intervention:**

In February 2021, Parkdale Queen West Community Health Centre, TELUS Health for Good, and University Health Network’s Gattuso Centre for Social Medicine partnered to launch a nurse practitioner‒led, community-based MHC in Toronto, Ontario. The MHC provides low-barrier primary healthcare, harm reduction, and mental health services at five convenient locations.

**Outcomes:**

Through an intercept survey (*n* = 49) and semi-structured interviews (*n* = 10), we sought to understand the sociodemographic characteristics of clients, their experiences at the MHC, and barriers and facilitators to the MHC in comparison to traditional healthcare settings. Most clients surveyed reported being homeless (61%). Without the MHC, 37% of clients would have accessed care at an emergency department and 18% would not have sought care. Thematic analysis revealed two structural and two relational factors that improved care experiences and care access.

**Implications:**

We demonstrate that in a single-payer health system, MHCs alleviate major barriers to care access for marginalized populations. Learnings provide context to the most salient factors influencing clients’ decisions to seek care at MHCs and can inform how these outreach models are designed.

## Résumé

**Lieu:**

Bien que le Canada ait un régime de santé à payeur unique, les populations marginalisées connaissent souvent une détérioration de leur santé et des obstacles à l’accès aux soins. Face à ce problème, des cliniques de santé mobile (CSM) ont été déployées dans plusieurs villes du pays. Les CSM sont bien établies aux États-Unis, mais on en sait peu sur leur rôle et leur incidence dans un pays doté d’un régime de santé universel. Nous décrivons la mise en œuvre d’une CSM urbaine et les premiers enseignements d’un processus à méthodes mixtes et d’une évaluation axée sur les résultats.

**Intervention:**

En février 2021, le centre de santé communautaire Parkdale Queen West, le programme Santé pour l’avenir TELUS et le Centre Gattuso pour la médecine sociale du Réseau universitaire de santé ont inauguré ensemble à Toronto une CSM communautaire dirigée par une infirmière praticienne. La clinique fournit des soins de santé primaires et des services de réduction des méfaits et de santé mentale «à bas seuil» dans cinq endroits faciles d’accès.

**Résultats:**

En interrogeant les gens au passage (n = 49) et en organisant des entretiens semi-directifs (n = 10), nous avons cherché à définir le profil sociodémographique des usagers, leur expérience de la CSM et les éléments qui entravent ou qui favorisent l’accès à la CSM comparativement aux milieux de soins classiques. La plupart des répondants (61%) ont dit être sans abri. Sans la CSM, 37% des usagers se seraient rendus aux urgences, et 18% ne se seraient pas fait soigner. Une analyse thématique a fait ressortir deux facteurs structurels et deux facteurs relationnels ayant amélioré l’expérience de soins et l’accès aux soins.

**Conséquences:**

Nous faisons la démonstration que dans un régime de santé à payeur unique, les CSM réduisent d’importants obstacles à l’accès aux soins pour les populations marginalisées. Nos constats mettent en contexte les principaux facteurs qui influencent la décision des usagers de se faire soigner dans les CSM; ils peuvent aussi éclairer la conception des modèles d’extension des services.

## Introduction

Despite Canada’s single-payer health system, socioeconomic disparities in health outcomes and healthcare access persist (Kitching et al., 2020; Kushel et al., 2001; Public Health Agency of Canada, 2018). In response to longstanding structural inequalities, there have been national calls for innovative approaches that address the social determinants of health (Canadian Institutes of Health Research, 2021; Public Health Agency of Canada, 2018). Mobile health clinics (MHCs) are an innovative model of care that have emerged as a community-driven response to address unmet health and social needs of marginalized populations. As a community-embedded intervention, MHCs are unique due to their mobility and nimbleness to rapidly evolve services and supports in response to needs. MHCs frequently support clients who are poorly served by the traditional healthcare system due to financial, geographic, and linguistic or cultural barriers (Yu et al., 2017). Additionally, some clients may prefer to access care at MHCs because of previous experiences with discrimination and stigma in traditional healthcare settings (Martins, 2008; Skosireva et al., 2014; Thornicroft et al., 2007; Wen et al., 2007).

MHCs are novel in the way they emphasize the interplay of space and place in the delivery of healthcare. Specifically, MHCs are often present in convenient locations and create an environment that blends traditional healthcare settings with a community feel. MHCs ensure that individuals who fall through the cracks of the traditional healthcare system get low-barrier access to timely prevention, screening, and support with system navigation (Attipoe-Dorcoo et al., 2020). Generally, MHCs offer a wide range of services using a trauma-informed, harm reduction, and client-centred approach to care. They may also act as a gateway to more traditional healthcare services through warm handoffs to community health centres, support with appointment booking, and referrals (Whelan et al., 2010).

In recent years, there has been a large increase in the number of MHCs in the United States, which has gained further traction due to barriers amplified by the COVID-19 pandemic (Attipoe-Dorcoo et al., 2020). Preliminary research suggests that MHCs have the potential to be cost-effective (Aung et al., 2015; Hill et al., 2014; Oriol et al., 2009), improve client experiences, and result in improved health outcomes (Edgerley et al., 2007). While MHCs are less common in Canada than in the USA, the number has increased substantially over the last decade. Based on an environmental scan conducted in 2022 (Nguyen et al., 2022), there were approximately 29 active primary care MHCs in Canada with the majority in Ontario, British Columbia, and Alberta. Despite widespread implementation across several provinces, there have been relatively few evaluations of MHCs conducted to date. Most evaluations have focused on specialized MHCs directed toward specific populations, such as Indigenous peoples (Oster et al., 2010; Virani et al., 2006) or people who use drugs (Lodge et al., 2022), or with a focus on the provision of specific services (Keboa et al., 2019).

### Description of Parkdale Queen West mobile health clinic

An evaluation of the TELUS Health for Good MHCs has occurred across five TELUS-funded clinics across Canada. The evaluation in the current study was informed by the approach taken at other TELUS-funded MHC sites in Nova Scotia, Ontario, and British Columbia. The Parkdale Queen West MHC (henceforth, the MHC) launched in December 2020 and is funded through the TELUS Health for Good initiative. The MHC is delivered by a nurse practitioner employed by a community-based organization (Parkdale Queen West) in partnership with a large academic hospital (University Health Network). During the height of the pandemic, the MHC primarily visited boarding homes and supportive housing settings to deliver COVID-19 vaccines. There was collaboration with Toronto Public Health for COVID-19 contract tracing, referrals, and transport of clients to COVID-19 isolation facilities. At present, the MHC serves approximately nine locations in downtown Toronto including shelters, community centres, high-density buildings, and encampments (Parkdale Queen West Community Health Centre, 2021). The MHC had three main objectives: (1) address unmet social and health needs through housing support, community navigation, emotional and crisis support, and food distribution, among others; (2) expand COVID-19 testing and vaccination; and (3) increase access to primary care and harm reduction services.

## Methods

### Participants

A convergent mixed methods design and outcome-oriented evaluation were conducted to understand the sociodemographics of clients and their experiences receiving healthcare at the MHC from July to August 2022. A convenience sample of clients was recruited by a Harm Reduction Coordinator to participate in an intercept survey (*n* = 49) and semi-structured interviews (*n* = 10) after receiving care from the MHC. This evaluation was deemed a quality improvement project as described by the Tri-Council Policy Statement V.2; thus, the study received approval waiver from the University Health Network.

### Data collection

The semi-structured interviews and participant intercept surveys were conducted by one Harm Reduction Coordinator in a private room, or in the office of a facility visited by the MHC. For the interviews, probes were used to elicit responses specific to evaluation questions. Each client interview was audio-recorded and transcribed verbatim by a professional transcription company. Interviews ranged from 8.2 to 30.3 min and lasted on average of 14.5 min. To preserve participant confidentiality, no identifying information was recorded. Fictitious names have been assigned to clients quoted in this paper. The interview guide and survey are available upon request.

### Coding and analysis

Interview transcripts were thematically analyzed by two members of the study team (MO, RR) (Miles et al., 2014; Patton, 2014). To enhance methodological rigour, both researchers coded the first two transcripts together to develop a draft coding structure, then the remainder of the transcripts were divided for independent coding. Coded transcripts were exchanged and meetings were held on an ad hoc basis to reach consensus and to ensure that emerging themes were reported concisely (Braun & Clarke, 2006). Coding was an iterative process that continued until no new themes emerged from the interviews. Transcripts were analyzed using NVivo 12 software.

## Results

Table 1 displays the demographic and health-related characteristics of MHC clients who participated in the intercept survey. More than half of the clients were 45 years and older (53.1%), with 40.8% identifying as an underserved ethnicity. The majority were male (71.4%), staying in a shelter (61.2%), and receiving financial aid (67.3%).

**Table 1:** Sociodemographic characteristics of clients (*n* = 49) who visited the Parkdale Queen West mobile health clinic

	***N* (%)**
Age group	
18‒34	15 (30.6%)
35‒44	7 (14.3%)
45‒54	12 (24.5%)
55 +	14 (28.6%)
Prefer not to answer/Don’t know	1 (2.0%)
Ethnicity	
Under-served	20 (40.8%)
White	26 (53.1%)
Don’t know/prefer not to answer	3 (6.1%)
Gender	
Male	35 (71.4%)
Female	11 (22.4%)
Prefer not to answer/Don’t know	< 5 (6.1%)
Living arrangement	
Not homeless	19 (38.8%)
Shelter	30 (61.2%)
Highest level of education	
Primary school (grade 1–8)	4 (8.2%)
Secondary or equivalent (grade 9–12)	17 (34.7%)
College	11 (22.4%)
University degree (bachelors and post-graduate)	8 (16.3%)
Prefer not to answer/Don’t know	9 (18.4%)
ODSP/OW recipient	33 (67.3%)
Prefer not to answer/Don’t know	3 (6.1%)
Total family income (before taxes)	
< $25,000	13 (68.4%)
≥ $25,000	2 (10.5%)
Prefer not to answer/Don’t know	4 (21.1%)
Born in Canada	29 (59.2%)
Health insurance status	
OHIP	45 (91.8%)
Other (e.g., Interim Federal Health, No insurance- OHIP eligible but no card, No insurance- non status, Private insurance)	4 (8.2%)
Physical well-being	
Excellent	6 (12.2%)
Very good	9 (18.4%)
Good	15 (30.6%)
Fair	13 (26.5%)
Poor	6 (12.2%)
Mental well-being	
Excellent	5 (10.2%)
Very good	9 (18.4%)
Good	13 (26.5%)
Fair	15 (30.6%)
Poor	6 (12.2%)
Prefer not to answer/Don’t know	1 (2.0%)

Table 2 characterizes client experiences and services received at the MHC. On average, clients visited the clinic three times, and highly rated the ease of accessing the clinic (9.2/10). The most common conditions addressed at the clinic were chronic conditions (27.1%), followed by mental health and substance use concerns (17.1%). The most common healthcare or referrals received were assessment or testing (35.6%) and treatment referral (30.7%). Clients highly rated the clinic’s preparedness and their relationship with staff (9.2 and 9.5 out of 10, respectively), while the wait times for MHC services were rated lower (8.6 out of 10). The majority of clients (55.1%) felt it was very important for the clinic to offer supports beyond health treatments, such as income assistance and finding shelter. If the mobile health clinic wasn’t available, the largest group of clients (36.7%) said they would seek care at the emergency department, while 18.4% would have received no medical care. A total of 18.4% of clients reported not having a usual source of care outside of the MHC. The most common reasons clients had not sought healthcare in the past were being too far from a provider or lacking transportation (28.2%) and not having enough time (15.4%).

**Table 2:** Client experiences (*n* = 49) and services received at the mobile health clinic

	*n* (%)
Number of times client has visited the mobile health clinic, mean (SD)	3.24 (2.9)
Number of times client has visited the mobile health clinic	
< 2 times	14 (28.6%)
2–3 times	19 (38.8%)
4–5 times	8 (16.3%)
6–7 times	5 (10.2%)
≥ 8 times	3 (6.1%)
On a scale of 1–10, how hard or easy is it to access care at the mobile health clinic? Mean (SD)	9.18 (1.42)
Health conditions addressed at the clinic	
Mental health and substance use	12 (17.1%)
Chronic conditions (e.g., diabetes, heart disease, high blood pressure)	19 (27.1%)
Infections (e.g., general wound care, upper respiratory infection)	7 (10.0%)
General pain	14 (20.0%)
Diabetic foot care	5 (7.1%)
Other (e.g., testosterone replacement therapy, transitioning support with gender-affirming surgery)	13 (18.6%)
Healthcare or referrals received^a^	
Assessment or testing	36 (35.6%)
Treatment referral (e.g., dental, vision, physiotherapy, psychotherapy referral)	31 (30.7%)
Prescription	25 (24.8%)
Social support (e.g., income/employment support)	3 (3.0%)
Substance use support or harm reduction (e.g., nicotine replacement therapy)	1 (1.0%)
Administrative support (e.g., access to medical records, obtaining identification)	3 (3.0%)
Other (e.g., support group for grief/loss)	2 (2.0%)
Client experiences of care	
On a scale of 1–10, how well prepared was the clinic?	9.16 (1.80)
On a scale of 1–10, how do you feel about the wait time for the mobile health clinic?	8.57 (2.05)
On a scale of 1–10, how do you feel about your relationship with mobile health clinic staff?	9.51 (1.00)
How important is it that the mobile health clinic offers additional supports, such as income assistance, transportation, and finding shelter, in addition to health treatments?	
Not very important	3 (6.1%)
Not important	3 (6.1%)
Important	13 (26.5%)
Very important	27 (55.1%)
Prefer not to answer/Don’t know	3 (6.1%)
Usual source of care outside of the MHC	
Clinic (e.g., walk-in clinic, community health centre)	8 (16.3%)
Family doctor	12 (24.5%)
Emergency at the hospital	18 (36.7%)
Nowhere	9 (18.4%)
Prefer not to answer/Don’t know	1 (2.0%)
Sources of care in the last 3 months	
Family doctor, but I no longer want to go there	8 (16.3%)
Family doctor, but I can no longer go there (e.g., too far, not in practice)	9 (18.4%)
Clinic (e.g., walk-in clinic, community health centre)	11 (22.4%)
Emergency at the hospital	16 (32.7%)
I have not sought care elsewhere	3 (6.1%)
Prefer not to answer/Don’t know	1 (2.0%)
Barriers to healthcare access^a^	
Too far/no transportation	22 (28.2%)
Not enough time	12 (15.4%)
Too stressed	11 (14.1%)
Lack of knowledge	3 (3.8%)
Can’t afford treatment/medication	6 (7.7%)
I don’t trust healthcare providers/I will feel judged	6 (7.7%)
Missing identification/insurance documents	7 (9.0%)
Language barriers	2 (2.6%)
COVID-19 pandemic	6 (7.7%)
Prefer not to answer/Don’t know	1 (1.3%)

^a^Select all that apply

### Relational themes

#### Staff enhance engagement through clear and unrushed communication

Participants expressed appreciation for the time that staff took to understand and hear their concerns and the use of simple and accessible language: “*I was treated with respect you know [the staff] were very patient and explained exactly what was going on. I wasn’t rushed in any way*” (Javier, returning client). Participants contrasted this to their experiences in traditional healthcare settings, where they felt rushed by doctors: “*[I come to the mobile health clinic] to renew my prescriptions. I have trouble with my stomach and my family doctor does not explain things very well because my doctor doesn’t have time for us. You know, he’s very busy*” (Taylor, returning client). Clients appreciated the directness of communication, with staff offering suggestions and recommendations for care. Participants noted that staff offered clear responses to their questions and used alternative methods to communicate when wait times for phone interpretation were long: “*Yeah, [staff at the mobile health clinic] use signs and pictures, so even though I am not fluent in English, they understand things and try to explain things with signs and body language*” (Juan, returning client). The staff encouraged clients to ask questions, which made them feel comfortable and reassured. The use of simple, direct, and a client-centred approach to communication helped clients understand their health and empowered them to make informed decisions about their healthcare.

#### Safe and inclusive environment at mobile health clinic promotes client comfort and equality

Clients were asked to provide a word or phrase that describes how they feel when they are at the MHC, which included feeling “*listened to*”, “*a sense of peace*”, “*helped*”, “*happy*”, “*good*”, “*comfortable and well taken care of*”, “*thankful that its there*”, “*confident*”, “*safe and comfortable*”, and “*a little anxious, but still on the positive side*”. One client mentioned the calm environment at the MHC in contrast with a visit to a hospital: “*The mobile health clinic means a sense of peace really. It’s not in the hospital atmosphere so it’s more like a little doctor’s office that’s more familiar, easier on the nerves*” (Xavier, returning client). Multiple clients expressed that they are always treated with dignity and respect: “*I go to the mobile health clinic because of the relationship. They assist me without discrimination, without looking down on me*” (Samantha, returning client). The strong relationship with MHC staff was also reflected in the overwhelmingly positive ratings in the intercept survey. Clients also appreciated the familiarity and consistency of the staff at the MHC: “*It’s more secure and safe to visit the mobile health clinic because when you go to a walk-in clinic, it’s a different person and personality where I may not know the person treating me. I feel happier visiting the mobile health clinic. I don’t want to go to walk-in clinics all the time*” (Anthony, first visit). Overall, clients valued the MHC for the familiar and safe environment that is cultivated, the provision of care that is non-judgemental, and the consistency of staff.

### Structural themes

#### Improving health and social care access by tackling logistical, financial, and administrative barriers effectively

Participants highlighted that the MHC is an important resource for care that is convenient, accessible, and reliable. Many participants appreciate the MHC’s frequent presence in various locations around the city, allowing them to overcome barriers related to costs and transportation issues. As one returning client noted, “*the benefit is that it’s right outside here you know? You don’t have to sit in a waiting room–it’s right there, it’s mobile. The weather doesn’t really stop you right? Snow or rain you’re going. Also, the people are kind and friendly*” (Malik, returning client).

To reduce financial barriers associated with transportation, the MHC offers transit passes. As another returning client explained, “*transportation takes so much of my money. But a few times I visited [the mobile health clinic] and [staff] gave me some tokens, which helped me a lot*” (Samantha, returning client). Transportation to care was also highlighted as the largest barrier for participants in the survey. Participants noted that the MHC is low barrier, as clients are not required to provide identification or a health card. One participant described the MHC as “*a clinic where people may get care easily and quickly without having [a] medical card and other documents. When I came here, I didn’t have anything. I do not have medical identification but I still [received] care. There is also [a] referral mechanism if things are beyond the mobile health clinic which is also helpful to newcomers or people living in shelters to access care quickly and easily*” (Juan, returning client).

Some participants have noted a sense of trust that is fostered by the MHC coming to them, rather than them seeking out care. As one first-time visitor to the MHC explained, “*I feel much safer and much [more] taken care of so I won’t hold back on my health issues. I don’t know how to describe it but first, [the mobile health clinic] comes [to us]. I don’t need to go out to look for the service so the convenience alone*” (Eva, first visit). The MHC’s accessibility and convenience have made it an important resource for many participants facing barriers to accessing healthcare.

#### Bridging the gap to health and social supports through system navigation assistance

MHCs can serve as an important initial contact point to the healthcare system, providing clients with system navigation assistance and building capacity for future in-person or virtual visits. Client examples demonstrate the range of health needs that this MHC addressed, including dental care, disability support, hormone replacement therapy, and access to identification, among others. For instance, a returning client shared their positive experience, saying “*well, I learnt today about asking for dental support and that they could help me get a dentist appointment*” (Malik, returning client). This is also the case in the intercept survey responses with nearly one third of clients receiving a referral or support with booking an appointment. Another client, on their first visit, was able to receive help with the Ontario Disability Support Program application: “*I told [staff] what kind of problems I have and that I want to apply to [the] Ontario Disability Support Program. I don’t know what forms to complete and then [staff] said we can work on it*” (Anthony, first visit). A returning client was referred to a specialist for hormone replacement therapy and surgery consultations: “*Since I am transgender, I take hormone replacement therapy and the [staff] sent me to Women’s College Hospital to see an Endocrinologist. I have another appointment to discuss surgery before I go in*” (Taylor, returning client). A first-time client was able to receive assistance in obtaining healthcare identification: “*The other thing he told me [is that] I should get a health card. Yeah, he advised that it should be arriving soon, so yeah, he supported me with getting access to ID and my health card*” (Eva, first visit). These examples highlight the value of this MHC in connecting clients to wider community resources through warm handoffs, support with appointment booking, and referrals.

## Discussion

This mixed methods evaluation describes the sociodemographic characteristics and experiences of clients who sought care at an urban MHC in Toronto, Ontario, Canada. The MHC offered a wide range of health and social supports including prevention, primary care, support with income assistance applications, wound care, and harm reduction supports, to name a few. We identified two relational and two structural factors that are significant to participants’ experience of care. Relational factors included an emphasis on taking time to understand clients and communicate care plans in an accessible way, as well as the provision of care in an inclusive environment. Structural factors included the effectiveness of the MHC in reducing logistical, financial, and administrative barriers to care and creating a bridge to health and social supports in the community through system navigation. This work highlights the potential for MHCs to complement the healthcare system as an equity-driven intervention to dismantle barriers to care.

MHCs are often thought of as an alternative to the traditional healthcare system; however, learnings from this evaluation reveal additional considerations. Clients in this study reported that the MHC served as an entry point for care in an inclusive and safe environment, connecting clients to the broader health system with appointments and referrals. Additionally, about 1 in 5 clients (18.4%) reported not having a usual source of care beyond the MHC, suggesting that MHCs are an important part of the overall health system. We observed that most clients were engaged and willing to access care that was beyond the scope of the MHC, in part due to the trusting relationships fostered by MHC staff, which is supported by the literature (Carmack, 2010; Hill et al., 2012; Rodriguez et al., 2007). Indeed, MHCs have the potential to play a complementary role to the broader health system through an intensive aim to reduce health inequities (Whelan et al., 2010). Furthermore, without the MHC, 37% of clients would have accessed care at an emergency department, suggesting that MHCs may play an important role in the provision of care in the most appropriate setting (Song et al., 2013). Given the ongoing strains faced in hospitals, interventions to direct clients to the most appropriate setting are of utmost importance.

MHCs also play a critical role in reducing transportation-related barriers, coordinating referrals to community agencies, addressing the social determinants of health through the provision of food and supplies, and supporting applications for social services. Attitudes about the healthcare system are shaped, in part, by previous healthcare encounters that influence an individual’s tendency to seek care (Park et al., 2021; Wen et al., 2007). The positive social aspects of their involvement can help clients overcome barriers to accessing traditional healthcare settings that arise from distrust of the system and prior experiences with stigma and discrimination (Martins, 2008; Skosireva et al., 2014; Thornicroft et al., 2007; Wen et al., 2007). Existing research that explored clients’ perceptions in other MHCs in Toronto has underscored the significance of trust building, equal treatment, and non-judgemental attitudes by staff (Daiski, 2005).

Across Canada, action on the social determinants of health is increasingly highlighted as a major health system goal (Canadian Institutes of Health Research, 2021). MHCs are one intervention that is well positioned to further these goals; however, further work is required to ensure they can sustainably serve the community. MHCs tend to be founded as pilot programs, relying on short-term funding from government or philanthropic sources (Yu et al., 2017). Moving beyond this is necessary to allow for long-term planning and to provide a solid foundation for the relationship-building capacity that MHCs offer. Cross-sectoral partnerships, as exemplified in this case, can also help with sustainability.

This study describes the sociodemographic characteristics and experiences of clients at a MHC, but it should be interpreted considering a few limitations. First, we interviewed a small convenience sample of participants that may not represent the broader experiences of all clients who receive care at the MHC. Although the Harm Reduction Coordinator conducting the interview was separate from the clients’ care team, it is possible that the presence of the interviewer may have biased responses more positively.

## Conclusion

This mixed methods evaluation explores the aspects of a mobile health clinic that are most valued by clients and highlights that even in a single-payer health system, MHCs play an important role in reducing barriers to care for marginalized populations. These learnings reveal the most influential factors that affect clients’ decision to seek care at MHCs and provide actionable insights on outreach interventions to address the social determinants of health.

## Implications for policy and practice

What are the innovations of this program?

- MHCs are an important intervention to consider when designing strategies to address health inequalities for marginalized populations that arise through structural barriers to healthcare.
- MHCs may function as an initial contact point into the broader healthcare system by supporting clients, especially those who may otherwise be disconnected or marginalized, with navigating the complexities of the system and building trust.

What are the burning research questions for this innovation?

- Although MHCs address health system goals related to action on health inequalities, the economic impacts of MHCs on the broader health system in Canada are unknown. While we observed evidence of divergence from emergency services because of access to the MHC, future work should seek to quantify this impact.
- Despite the benefits of MHCs in reaching marginalized populations, they are often viewed as distinct from mainstream healthcare. Future research should explore the barriers and enablers for the integration of MHCs into the broader healthcare system.

## Data Availability

The data generated and analyzed during the current study are available from the corresponding author upon reasonable request and with Research Ethics Boards approval.
